# Encéphalopathie pancréatique: à propos de deux cas

**DOI:** 10.11604/pamj.2016.25.147.9324

**Published:** 2016-11-11

**Authors:** Nawfal Doghmi, Aziz Benakrout, Amine Meskine, Mustaphja Bensghir, Abdelouah Baite, Charki Haimeur

**Affiliations:** 1 Pôle d’Anesthésie Réanimation, Hôpital Militaire d’Instruction Mohamed V Rabat, Université Souissi Mohamed V Rabat, Maroc

**Keywords:** Tumeur embryonnaire, lymphome, chimiothérapie, Severe acute pancreatitis, post-pancreatic encephalopathy, axonal damages

## Abstract

L'encéphalopathie pancréatique, est une complication rare de la pancréatite aiguë, notre étude porte sur 02 cas d'encéphalopathie pancréatique, hospitalisés et traités au sein du service de Réanimation chirurgicale de l'Hôpital Militaire d'Instruction Mohamed V de Rabat. L'âge des patients était compris entre 43 ans et 54 ans, nos 02 cas sont repartis en une femme et un homme. Le mécanisme physiopathologique de l'EP n'est pas encore bien élucidé, de nombreuses hypothèses ont été rapportées dans la littérature, certains auteurs suggèrent que la lipase et la Phospholipase A2 jouent un rôle dans le processus pathologique de l'EP. D'autres facteurs tels que les infections, les troubles hydroélectrolytiques, l'hypoxémie et la perturbation de la glycémie, peuvent être déclencheurs. Le diagnostic de l'encéphalopathie pancréatique est facile à établir, la symptomatologie clinique se résume le plus souvent à une confusion, avec stupeur, et agitation psychomotrice, Il s'y ajoute parfois des atteintes neurologiques comme des convulsions, une céphalée, des hémiparésies passagères, une dysarthrie, enfin des difficultés d'expression verbale et une amnésie. Les examens paracliniques, notamment L'IRM cérébrale et l'électroencéphalogramme, permettent de confirmer le diagnostic. Le traitement est d'abord symptomatique, il a comme objectif de lutter contre les facteurs qui favorisent l'apparition des signes neurologiques par les mesures de réanimation que réclame la gravité de la situation. L'évolution de l'EP est le plus souvent favorable, avec une disparition progressive des symptômes, cependant la persistance de quelques séquelles, est décrite dans la littérature. Le pronostic est fonction de la gravité de la pancréatite aigüe et des complications associées. Dans notre étude les données sont globalement comparables à celles publiées actuellement par la majorité des auteurs.

## Abstract (en)

Pancreatic encephalopathy (PE) is a rare complication of acute pancreatitis. Our study reports 2 cases of patients with pancreatic encephalopathy, hospitalized and treated in the Intensive Care Unit of the Military Hospital of Instruction Mohammed V, Rabat. Patient age ranged between 43 and 54 years, our 2 cases involved a woman and a man. The pathophysiologic process of EP is still not well understood, many assumptions have been described in the literature; some authors have suggested that lipase and phospholipase A2 are involved in the pathological process of PE. Other factors including infections, fluid and electrolyte disturbances, hypoxemia and perturbations in blood glucose can be triggers. The diagnosis of pancreatic encephalopathy is easy to establish, clinical symptoms usually include confusion, amazement and psychomotor agitation, sometimes associated with neurological damages such as convulsions, headache, transient hemiparesis, dysarthria, difficulties in verbal expression and amnesia. Paraclinical tests, including brain MRI and electroencephalogram allow a definitive diagnosis. Treatment is primarily symptomatic aiming to fight against factors favoring the onset of neurologic signs using resuscitative measures based on severity of the situation. The prognosis depends on the severity of acute pancreatitis and its complications. In our study data are broadly comparable to those currently published by the majority of authors.

## Introduction

L'encéphalopathie pancréatique(EP), est une complication rare de la pancréatite aiguë grave (PAG), qui a été décrite pour la première fois en 1941 par Rothermich et Coll [1] et plus tard par Vogel [2]. Elle est caractérisée par une association des signes neurologiques qui peuvent se produire au cours des deux premières semaines de la pancréatite aiguë, quel que soit son étiologie. Actuellement, il n'y a pas d'examens spécifiques ou unifié à des normes concernant le diagnostic des encéphalopathies pancréatiques. Le diagnostic repose sur les signes cliniques ou les antécédents de pancréatite aiguë associés à des symptômes neuropsychiatriques [3]. L'apport des examens complémentaires reste limité. L'examen anatomopathologique montre une démyélinisation cérébrale diffuse. L'Imagerie par résonance magnétique (IRM) montre des signaux anormaux au niveau de la substance blanche [4]. Les rapports mondiaux sur l'encéphalopathie pancréatique ont augmenté récemment mais sa pathogénie reste incertaine [5]. Nous rapportons dans ce travail 02 cas d'encéphalopathie pancréatique, colligés au service de réanimation chirurgicale de l'Hôpital Militaire d'Instruction Mohamed V.

## Patient et observation

### Observation 1

**Figure 1:**
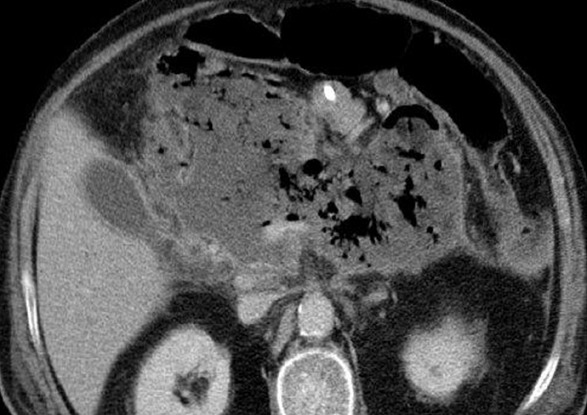
TDM abdominale C+ pancréatite avec nécrose infectée

**Figure 2:**
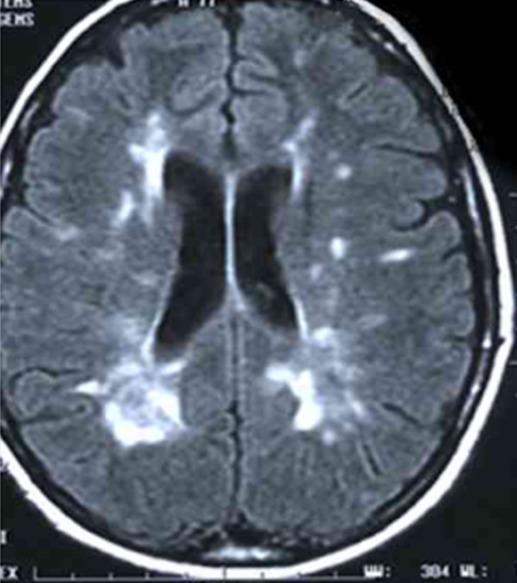
IRM cérébrale: présence d’hyper signaux dans la substance blanche en T2

Il s'agissait d'un patient âgé de 54 ans, suivi pour hypertension artérielle et hypertriglycéridemie, admis au service de réanimation pour pancréatite aigue grave. A son admission on a noté un syndrome douloureux abdominale isolé, l'état général était conservé, le patient était stable sur le plan hémodynamique. Avec une Tension artérielle à 130 /80 mmhg, une Spo2 à 98% à l'air ambiant, patient était conscient bien orienté dans le temps et l'espace avec un score de Glasgow coté à 15/15, la température était à 37°C, son abdomen était souple, transit audible, et le toucher rectal était sans anomalie. Le bilan biologique montrait une hyperleucocytose à 20000/mm^3^ une hématocrite à 27%, une insuffisance rénale avec une urée à 0,71 g/l, une Créatinine à 35 g/l, une lipasémie à 20000 UI/l, le bilan lipidique était perturbé avec augmentation de triglycérides, de HDL et de LDL, le Bilan hépatique montre des ASAT à 50 UI/l, ALAT à 35 UI /l, Bilirubine totale à 10 UI/l, la CRP était à 50 mg/l. L'ECG montrait une tachycardie isolée à 102 cycles/min, avec bloc de branche droit, et absence de signes d'ischémie, la Troponine était normale et la radiographie pulmonaire montrait une minime réaction pleurale gauche. L'échographie abdominale objectivait une lithiase vésiculaire, les voies biliaires intra et extra hépatiques étaient libres. La TDM abdominale avec injection a montré une pancréatite aigue avec coulée péri pancréatique et para rénale. Le diagnostic d'une pancréatite aigue grave stade E était retenu avec score de Ranson à 4 et APPACHE II à 15. Après mise en condition du patient: réhydratation par du sérum salé 0.9% 500 cc/08h et sérum glucosé 5% 500 cc /12h avec apport des électrolytes, oxygénothérapie par lunettes à O2, sonde nasogastrique avec jeune strict, matelas antiescares avec prévention de la maladie thromboembolique et de l'ulcère de stress. Le traitement initial était symptomatique avec maintien de l'hémodynamique guidé par la Pression artérielle moyenne et la diurèse, une analgésie par le Nefopam et la morphine par PCA morphine. A J+8 le patient a présenté brutalement une hyperthermie à 39°c associée à un tableau neurologique fait de délire, des hallucinations visuelles et une dysarthrie, Compliqué d'agitation psychomotrice, ce qui a justifié le cours à des sédatifs à base de neuroleptiques, benzodiazépines. Le bilan réalisé montrait une hyperleucocytose à 30.000/mm^3^; une CRP à 230 mg/l; une thrombopénie a 80.000 éléments/mm^3^, avec une hypophosphatémie, hypomagnesémie. La TDM abdominale était en faveur d'une nécrose infectée (Figure 1) et l'IRM cérébrale montrait des lésions diffuses de la substance blanche (Figure 2 ). Le patient était mis sous sédatifs à base de neuroleptiques et benzodiazépines, antalgiques, une Antibiothérapie par l'Imipenème 500mg/6h en IVL et une Supplémentation de magnésium et de phosphore. A J+11, l'évolution était favorable, le patient est devenu calme, apyrétique avec des leucocytes à 7000 et une CRP à 120 mg/l. A j+15 l'alimentation orale est autorisée progressivement. A j+18: l'IRM cérébrale de contrôle montrait une régression des lésions initiales. Puis le patient était transféré au service de gastro-entérologie avec comme séquelle une amnésie partielle et une discrète dysarthrie.

### Observation 2

Il s'agissait d'une femme âgée de 43 ans, hypertendue depuis 5 ans sous diurétique et régime sans sel, hospitalisée au service de réanimation pour pancréatite aigue évolutive. L'examen clinique trouvait une patiente consciente, sans déficit sensitivomoteur, elle accuse une douleur abdominale transfixiante isolée, toutefois l'abdomen était souple, une tension artérielle à 140/70 mmHg, une fréquence cardiaque à 100 battement/min, une température à 37,2°C, une SpO2 à 98% à l'air libre. Le bilan biologique initial montrait une hyperleucocytose à 14.000 éléments /mm^3^, une hémoglobine à 11 g/dl, des Plaquettes à 241.000 éléments /mm^3^, une Glycémie à 2,42 g/l, une hypocalcémie à 45 mmol/l, une amylasémie à 1270 UI/l, une lipasémie à 3500 UI/l, une CRP à 172 mg/l. Le bilan hépatique et de l'hémostase étaient sans anomalies, Le scanner thoraco-abdominal avec injection a montré une pancréatite aigue stade E sans anomalies sur les voies biliaires avec un épanchement pleural gauche de moyenne abondance. Le diagnostic d'une pancréatite aigue grave stade E était retenu avec un score de Ranson à 03 et APPACHE II à 10. Après mise en condition de la patiente, réhydratation, oxygénothérapie, matelas anti escarres. Le traitement s'est basé sur l'optimisation de l'hémodynamique, l'insulinothérapie à la seringue électrique, l'analgésie parentérale associant du Paracétamol et du Nefopam, la Prévention de la maladie ulcéreuse et thromboembolique. Une alimentation parentérale sur une base de 2400 Kcal/j, un traitement antihypertenseur par Nicardipine retard 50 mg/j et des soins de nursing. A J+10, la patiente a présenté brutalement une détresse respiratoire aigue avec polypnée, une SpO2 à 75% Sous masque à haute concentration, une température chiffrée à 37.5°C. La gazométrie montrait une SaO2 à 65%, une PaO2 à 45mmHg, la radiographie thoracique montrait un épanchement pleural gauche de grande abondance qui a été drainé par un drain thoracique (Joly 24) et la patiente a été mise sous ventilation non invasive. Par la suite on a noté une amélioration de l'hématose, une SpO2 à 96% sous lunettes. La Gazométrie de contrôle était sans anomalies. A j+12 la patiente a présenté une confusion mentale avec désorientation temporo-spatial, des scotomes puis une agitation qui a régressé rapidement sous Hypnovel et hydroxyzine. Le scanner cérébral réalisé après avoir jugulé l'agitation était sans anomalies. L'EEG montrait des ondes thêta lentes. Le Bilan ionique et phospho calcique était normal avec absence d'hypomagnésemie. Le scanner abdominal montrait la persistance des coulées sans signes d'infection. La patiente était mise sous Magnésium avec complexes vitaminiques. L'évolution était favorable sans séquelles. On a autorisé l'alimentation orale à j+15 puis la patiente était transférée au service de gastroentérologie à j+20. Le diagnostic retenu était une pancréatite aigue grave compliquée d'une encéphalopathie secondaire à une hypoxie sévère ayant favorablement évoluée.

## Discussion

L’encéphalopathie pancréatique est une entité rare, sa fréquence reste variable selon les auteurs. Selon plusieurs études elle varie entre 4% et 18,2% [5, 6]. Au service de réanimation chirurgicale de l’Hôpital Militaire d’Instruction Med V, sur une période de 05 ans (2006 - 2010), 02 cas d’EP ont été prise en charge, sur 40 cas de PAG hospitalisées. Ce qui donne une fréquence de 5% (donnée non publiée). Dans de nombreuses études l’âge moyen de survenue de l’encéphalopathie pancréatique, varie entre 47 ans et 49 ans [4, 5]. Dans les 02 cas rapportés dans notre travail, l’âge des Patients était de 43 ans et 54 ans. La répartition selon le sexe est très variable, dans des séries les hommes sont plus nombreux que les femmes, alors que dans d’autres séries on note une prédominance féminine. Dans la série de Sun Bei et coll. [6] qui compte 21 cas d’EP, 11 cas étaient des hommes et 10 étaient de sexe féminin. On note alors une faible prédominance masculine. Une autre série de DING X et coll. [5] incluant 132 patients hospitalisés pour la prise en charge d’une PAG, 24 patients ont été compliqués d’EP. Parmi ces 24 patients Onze étaient des hommes et 13 des femmes, ce qui montre une faible prédominance du sexe féminin. Le mécanisme physiopathologique de l’EP n’est pas encore bien élucidé, de nombreuses hypothèses ont été rapportées dans la littérature.Les remaniements pathologiques dans les tissus cérébraux pendant l’encéphalopathie pancréatique sont principalement l’œdème toxique des neurones, avec la présence des foyers hémorragiques [7]. Des études récentes ont trouvé plusieurs facteurs lies à la pathogenèse des encéphalopathies pancréatiques et aucun facteur unique ne peut expliquer entièrement la cause [8]. Certains auteurs [9] suggèrent que la lipase, la Phospholipase A 2 (PLA2) et les cytokines peuvent endommager les structures du système nerveux central, et jouent un rôle dans le processus pathologique de l’EP. La PLA2 activée transforme l’enképhaline et la lécithine en enképhaline cytotoxiques hémolytique, et en lécithine hémolytique. Qui peuvent endommager la barrière hémato-encéphalique, dissoudre la structure phospholipidique de la membrane neuronale, hydrolyser les mitochondries, causant ainsi un trouble du métabolisme cérébral et un œdème , ce qui provoque une démyélinisation sévère dans les axones et des dommages au niveau des vésicules d´acétylcholine, inhibant ainsi la libération d´acétylcholine et aboutissant à la perturbation de la transmission neuromusculaire. Egalement Les cytokinesComme le TNF-alpha, IL-1beta et l´IL-6 endommagent la barrière hémato-encéphalique, augmentant ainsi sa perméabilité, ce qui déclenche l´activation et l’agrégation des leucocytes, avec des lésions neuronales et endothéliales directes responsable des remaniements inflammatoires de la gaine de myéline [9]. D’autres facteurs tels que l’hyponatrémie, l’hypophosphorémie, le manque en magnésium et en vitamine B1, l’hypoxémie, la perturbation de la glycémie et l’infection peuvent être déclencheurs [2, 10]. Dans notre étude pour le 1^er^ cas, le facteur déclencheur était l’infection de la nécrose, associé à une hypomagnésémie, et une hypophosphorémie. Concernant le 2 cas, le facteur déclencheur était l’hypoxémie secondaire à la détresse respiratoire. Ces facteurs sont en accord avec les données de la littérature. Le diagnostic de l’EP est essentiellement clinique, l’installation de la symptomatologie peut survenir brutalement avec apparition de convulsions, une amaurose (en raison d´une névrite optique aiguë ou à une rétinopathie hémorragique), une parésie d´un ou de plusieurs membres, une dysarthrie comme elle peut avoir un début progressif avec des changements de comportement, une agitation psychomotrice, une désorientation dans le temps et l´espace, et des hallucinations visuelles et auditives sur un état d´obnubilation qui peut arriver au stade de coma [11]. Le délai d’apparition des signes neurologiques, varie selon les cas décrits dans la littérature, L’étude de DING X et coll. Rapporte un délai allant de 3 heures jusqu’à 38 jours avec une moyenne de 6,6 jours. Sur 24 patients inclus, 21 patients (soit 87,5%) ont manifesté les signes dans les 2 semaines et 3 patients (soit 12,5%) après deux semaines [5]. Dans notre travail, le tableau clinique révélateur de l’EP est conforme aux tableaux décrits dans la littérature et le délai d’apparition des signes était moins de 02 semaines, ce qui est proche aux données de la littérature. L´Imagerie par résonnance magnétique (IRM) cérébrale est l’examen de choix dans le diagnostic positive de l’EP, elle est précocement indiquée, même si elle n´a pas d’effet sur la prise en charge, et elle montre des lésions bilatérales diffuses de la substance blanche [3]. L’électroencéphalogramme montre des signes peu spécifiques (ondes thêta ou delta), avec une activité lente sans décharge paroxystique [1, 12]. Ces données concordent avec celles de nos 02 observations. L’encéphalopathie pancréatique est une complication grave de la pancréatite aiguë grave, le temps de déclenchement est court, caractérisée par une mortalité élevée d’ou l’intérêt de la prévention, le diagnostic précoce, et la prise en charge adéquate peut réduire sa gravité. Par conséquent le traitement initial des complications est la clé pour prévenir l’encéphalopathie pancréatique [13]. Le traitement préventif consiste à la correction des troubles hydro électrolytiques, la surveillance de l’état hémodynamique, l’état respiratoire, de la saturation en O2 en assurant l’approvisionnement des tissus en O2 par une oxygénothérapie nasale et en maintenant les voies respiratoires perméables par l’évacuation des épanchements et la kinésithérapie respiratoire qui permettent d’éviter l’intubation et la ventilation mécanique, également la surveillance de la température avec lutte contre l’infection, par l’instauration d’une antibiothérapie prophylactique et/ou curative [14]. L’utilisation de certains inhibiteurs de la trypsine tels que la somatostatine, et l’UTI (Urinary Trypsin Inhibitor) [15] peut réduire la surcharge sur le pancréas, diminuer un des causes de l’EP, et permettre de soulager les symptômes. Lorsque les patients présentent des symptômes neuropsychiatriques, le recours aux sédatifs et aux neuroleptiques est justifié [13]. La lutte contre les autres facteurs qui favorisent l’apparition des signes neurologiques, comme la carence en vitamine B1 [2, 16] et les troubles hydro électrolytiques surtout l’hypomagnésémie et l’hypophosphorémie [17, 18] et les troubles métaboliques comme l’hyperglycémie [11] est indispensable. Dans notre travail, la prise en charge des 02 patients était conforme aux recommandations de la littérature. L’évolution de l’EP peut être favorable, avec une disparition progressive des symptômes, cependant la persistance de quelques séquelles comme les troubles cognitives, l’apathie, et la réduction de la fluidité verbale sont décrits dans la littérature [19]. Chez nos 02 patients, l’évolution était favorable, sans séquelles chez le 1^er^ patient, cependant la 2^ème^ patiente a gardé comme séquelles une amnésie partielle et une discrète dysarthrie. Bien que le taux de guérison des pancréatites aigues graves a augmenté de façon marquée, 18,2% des patients ont encore la complication de l’encéphalopathie pancréatique. Etant donné que les taux de mortalité est de 67,0%. L’encéphalopathie pancréatique reste un facteur de mortalité élevée [6, 20].

## Conclusion

L'encéphalopathie pancréatique est une affection rare, de pronostic redoutable. Son mécanisme physiopathologique n'est pas encore clarifié malgré toutes les théories proposées. Les étiologies de cette affection sont nombreuses mais dominées par les infections, les troubles hydro électrolytiques et l'hypoxémie secondaire aux PAG. Le tableau clinique, l'IRM cérébrale, et l'EEG, permettent le diagnostic positif précoce, et la prise en charge rapide. Le pronostic de l'EP dépend du dégrée de la pancréatite, et par conséquent le traitement initial des complications est la clé pour prévenir l'encéphalopathie.
